# Ultraviolet-B Wavelengths Regulate Changes in UV Absorption of Cleaner Fish *Labroides dimidiatus* Mucus

**DOI:** 10.1371/journal.pone.0078527

**Published:** 2013-10-15

**Authors:** Jill P. Zamzow, Ulrike E. Siebeck, Maxi J. Eckes, Alexandra S. Grutter

**Affiliations:** 1 School of Biological Sciences, The University of Queensland, Brisbane, Queensland, Australia; 2 School of Biomedical Sciences, The University of Queensland, Brisbane, Queensland, Australia; The Australian National University, Australia

## Abstract

High-energy wavelengths in the ultraviolet-B (UVB, 280-315 nm) and the UVA (315-400-nm) portion of the spectrum are harmful to terrestrial and aquatic organisms. Interestingly, UVA is also involved in the repair of UV induced damage. Organisms living in shallow coral reef environments possess UV absorbing compounds, such as mycosporine-like amino acids, to protect them from UV radiation. While it has been demonstrated that exposure to UV (280-400 nm) affects the UV absorbance of fish mucus, whether the effects of UV exposure vary between UVB and UVA wavelengths is not known. Therefore, we investigated whether the UVB, UVA, or photosynthetically active radiation (PAR, 400-700 nm) portions of the spectrum affected the UV absorbance of epithelial mucus and Fulton’s body condition index of the cleaner fish *Labroides dimidiatus*. We also compared field-measured UV absorbance with laboratory based high-performance liquid chromatography measurements of mycosporine-like amino acid concentrations. After 1 week, we found that the UV absorbance of epithelial mucus was higher in the UVB+UVA+PAR treatment compared with the UVA+PAR and PAR only treatments; after 2 and 3 weeks, however, differences between treatments were not detected. After 3 weeks, Fulton’s body condition index was lower for fish in the UVB+UVA+PAR compared with PAR and UVA+PAR treatments; furthermore, all experimentally treated fish had a lower Fulton’s body condition index than did freshly caught fish. Finally, we found a decrease with depth in the UV absorbance of mucus of wild-caught fish. This study suggests that the increase in UV absorbance of fish mucus in response to increased overall UV levels is a function of the UVB portion of the spectrum. This has important implications for the ability of cleaner fish and other fishes to adjust their mucus UV protection in response to variations in environmental UV exposure.

## Introduction

 Ultraviolet (UV) radiation is damaging to living tissues (e.g. [1]). The high-energy wavelengths in the UVB portion (UVB, 280-315 nm, International Commission on Illumination or Commission Internationale de L’Eclairage, C.I.E.) of the UV spectrum are directly absorbed by DNA and are particularly damaging [2-4]. While the lower-energy UVA (315-400 nm, C.I.E.) portion of the spectrum is detrimental via the actions of reactive oxygen species, such as singlet oxygen generated by photosensitizers [3,5], it also contributes to photo-activated repair of UVB-induced cellular damage [5,6]. 

In and near the tropics (0°-30° latitude), more UV radiation (UVR) reaches the Earth than in temperate regions, due to a lower zenith angle and a shorter light path [7,8]. As a consequence, tropical regions experience the highest doses of UVR [9] and overall solar radiation [10] on the planet. Furthermore, small but statistically significant increases in yearly solar irradiance levels have been demonstrated for our study site in Queensland, Australia [11]. Organisms living in coral reef environments are vulnerable to solar UV radiation as their shallow habitats are high in UV radiation [7,12-14]. Recently, melanoma was found in the coral trout, a predatory reef fish species [15], demonstrating that UVB may be problematic in tropical aquatic habitats if UV levels continue to increase.

 Coral reef fishes have UV absorbing compounds in the corneas, lenses, and humors of their eyes as well as in their epithelial mucus [16,17]. The UV absorbing compounds in fish epithelial mucus have recently been identified as mycosporine-like amino acids (MAAs; [18]), which are acquired from the diet [19,20]. The ability of fish epithelial mucus to absorb UV radiation varies considerably among species [17,18], and correlates with latitude [18,21], water clarity [22] and depth of capture [17,23]. 

 When provided with MAAs in their diet, the mucus of experimental fish that were exposed to UV had a much higher UV absorbance than the mucus of fish that were protected from UV exposure [20]. This shows that exposure to UV affects UV absorbance in fish mucus. It also suggests that the sequestration of MAAs may be energetically costly and may only occur when necessary. Whether exposure to the shorter-wavelength UVB compared with the longer-wavelength UVA affects the absorbance of mucus differently, however, has never been tested. UV levels in seawater decrease with increasing depth [14,24]. Insofar as they function as UV screening compounds, it is no surprise that MAA concentrations in macroalgae, corals, and some other invertebrates decrease with depth [25-28]. Zamzow and Losey [17] found that fish from the comparatively turbid waters of Kaneohe Bay, Hawaii possessed less absorbent mucus with increasing depth. To our knowledge, however, this relationship has not been examined for clear waters such as those of Lizard Island, Great Barrier Reef (GBR), Australia. Clearer waters transmit higher UV radiation levels, which has been shown to result in fish possessing higher concentrations of UV screening compounds in their mucus compared to fish from more turbid waters at comparable depths [22].

 Cleaner fish *Labroides dimidiatus* eat parasites and epithelial mucus from client fish on coral reefs [29] and are thought to be important players in reef ecosystems [30,31]. *L. dimidiatus* in shallow water, however, are likely subject to high levels of UV exposure as they spend most of their time in the open, cleaning other fishes and displaying to attract client fish [32,33]. Indeed, cleaner fish have been shown to have very high levels of MAAs in their mucus [18]. Here, we used cleaner fish as a model system to investigate whether the UVB, UVA or photosynthetically active radiation (PAR, 400-700 nm, C.I.E.) portions of the spectrum contributed to changes in epithelial mucus absorbance and Fulton’s body condition index (fish weight/fish standard length^3^, [34]). 

 We compared both epithelial mucus absorbance and Fulton’s body condition index of cleaner fish during, and after, 3 weeks of exposure to one of three treatments: 1) UVB+UVA+PAR, 2) UVA+PAR, or 3) PAR only. Epithelial mucus absorbance was measured using high-performance liquid chromatography (HPLC) as well as field spectrometry to validate the much cheaper and quicker method of field spectrometry. In a separate experiment, we assessed wild-caught fish from a range of depths to determine if depth of capture was correlated with the ability of epithelial mucus to absorb UV radiation. 

## Materials and Methods

### Ethics statement

All experiments were conducted according to the Australian code of practice for the care and use of animals for scientific purposes. The protocol was approved by the Animal Ethics committee of The University of Queensland (permit: ZOO/ENT/661/04/UQFREA).

### Fish collections

 Fieldwork was performed at Lizard Island Research Station (14°41’S, 145°27’E) between November and December of 2005. Cleaner fish were collected from depths of 3 to 16 m by SCUBA divers with barrier and hand nets (collection permits GBRMPA GO4/12405.1 & GO4/12017.1 and Fisheries #PRM02841J). All cleaner fish used in the spectral experiment were collected from ≤ 3 m. These fish were initially transferred to holding tanks exposed to full sunlight, they were then sampled indoors for mucus absorbance, and transferred to experimental tanks outdoors where they were held for the duration of the experiment; the final transfer occurred within 4 h of capture. Fish were fed daily throughout the experiment on a diet of mashed prawns mixed with commercial flake food (OSI, Hayward, CA, USA); a HPLC analysis of this diet was performed to determine its MAA content. Tanks were cleaned at least every 5 d to minimize the presence of biofouling organisms that might be eaten by the cleaner fish. 

### Spectral experiment

**Figure 1:**
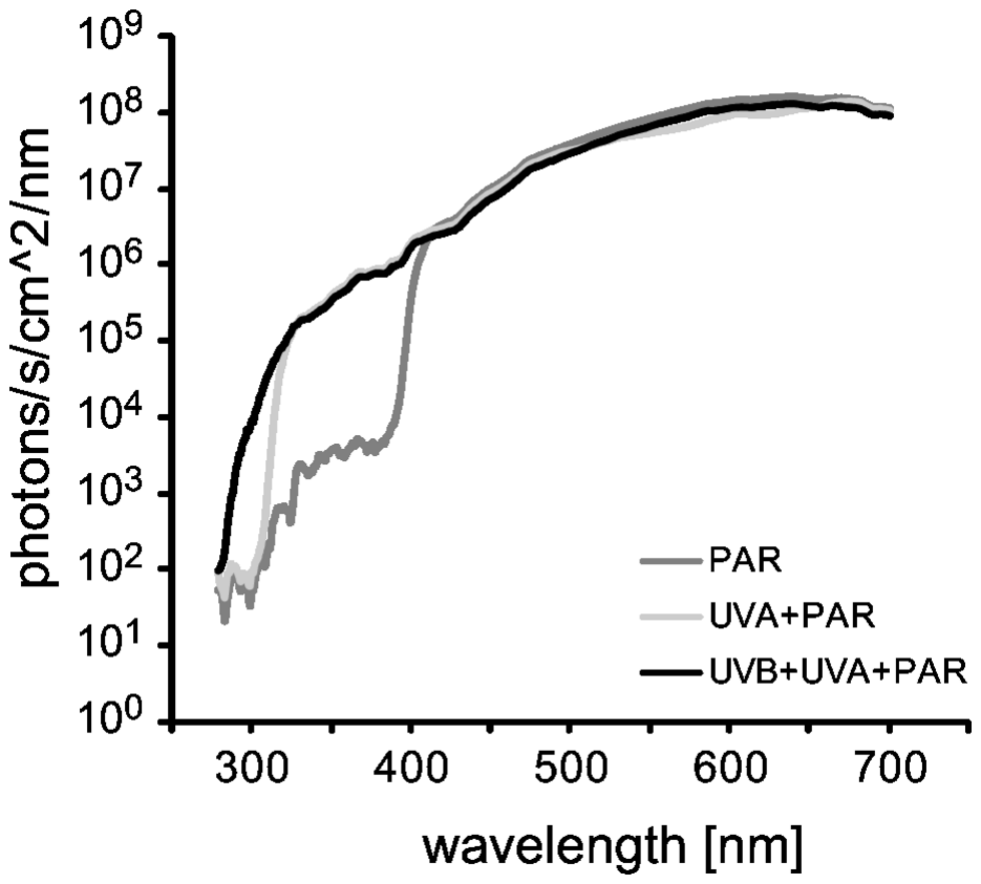
Radiant energy distribution of the three treatments used in the spectral experiments. PAR = photosynthetically active radiation, UVA = ultraviolet-A, and UVB = ultraviolet-B.

To test the effect of different light spectra on the mucus absorbance of the fish, we exposed fish to either PAR alone, PAR & UVA or PAR, UVA and UVB (Figure 1). Thirty-six experimental aquaria (33.5 cm L x 23.5 cm W x 27 cm H) with flow-through seawater and aeration were exposed to full solar radiation. Each aquarium housed one fish, which was randomly assigned to one of three spectral treatments. The desired spectral exposures were obtained via aquarium lids comprised of acrylic (Acrylite, Cyro Industries, New Jersey, USA) and filters (Lee Filters, U.K.). The UVB+UVA+PAR treatment included all wavelengths from 280 to 700 nm (Cyro Acrylite OP-4 plus Lee Filters #HT 254), the UVA+PAR treatment included wavelengths from 315-700 nm (Lee Filters #053), and the PAR treatment included wavelengths from 400-700 nm (Cyro Acrylite OP-3). The sides of all aquaria were covered with diffuse filters (Lee Filters 129) that transmitted approximately 5% of available PAR but did not transmit UVB or UVA. 

Mucus from each fish was sampled within 4 h of capture, and again weekly for three weeks, as described in Zamzow and Losey [17]. Briefly, the flank of each fish was scraped with a dull scalpel blade, collected mucus was squashed to 0.25 mm between two UV-transparent slides, and UV absorbance was measured with a fiber-optic spectrometer (S-2000, Ocean Optics, FL, USA). Eight absorbance measurements were taken of each sample, and the mean spectrum was used for data analysis. Following this spectrometry, mucus samples from weeks two and three were preserved in liquid nitrogen and transported to The University of Queensland for later analysis by HPLC. 

For each fish, the area under the UV absorbance curve was integrated as in Zamzow [20] and the percent change in mucus absorbance over time was calculated. The percent change in integrated UV absorbance of the mucus was analyzed via repeated measures ANOVA with a first-order autoregressive covariance structure, Satterthwaite determination of degrees of freedom, and Bonferroni adjusted pair wise t-tests for differences between treatments within each week (SAS v 9.2, SAS Systems Inc., NC, USA). 

 At the end of the spectral experiments, each fish was measured (total length, TL) to the nearest 0.1 cm, and weighed by measuring the amount of water the fish displaced to 0.1 g (weight, W). Fulton’s condition index, K was calculated for each fish as K = W/TL^3^ [34]. K values were compared, pair wise, between treatments and against wild-caught fish from the same area and depth range.

To assess the effect of duration in captivity on UV absorbance, fish (n = 35) were captured from ≤ 3 m depths. Each fish was randomly assigned a duration in captivity which ranged from 2 to 14 days, and fish were housed under the UVB+UVA+PAR treatment. Initial and final mucus samples were taken and analyzed as above.

### Effect of reef depth

 For the depth study, cleaner fish (n = 61) were collected from depths ranging between 3 and 16 m. The deepest depth reached on each collection dive was assigned as the capture depth. Range of depths on a single dive generally varied no more than 2 m, except in the case of a single 9 m dive, which was on a steep slope and capture depth varied over 6 m. A single mucus sample was taken from each fish as described above and analysed with field-spectrometry only.

### HPLC analysis

HPLC is often used to measure the MAA content of animals and plants [35-39], but we examined whether the simpler, less expensive, and less time-consuming method of spectrophotometry is equally valid for measurements of bulk MAA absorption, and whether that absorption correlates with actual MAA concentration. HPLC analysis was performed at The University of Queensland from August 2007 to January 2008. The methods for mucus extraction and dry weight quantification followed those of Eckes et al. [18]. Mucus extracts were suspended in 500 μl of MilliQ water and passed through a Millex 0.22 μm syringe-driven filter (Millipore, North Ryde, Australia). Filtered samples were added to new glass-shell vials (Waters Corp, Milford, Massachusetts) and 2 μl of each sample was injected into a liquid chromatograph system (Shimadzu LC-10AT VP, Eagle Farm, Australia). Samples were further diluted with MilliQ water if absorbance was saturated or peaks did not saturate (about 10% of the time). MAAs were eluted through a Devenosil RPAQEOUS Column (Phenomenex, Pennant Hills, Australia) using a gradient from an aqueous mobile phase of 0.05% aqueous formic acid to 0.05% formic acid in 100% methanol at a flow rate of 250 μl min^-1^ (Table 1). Peaks were detected using a photo diode array detector (Shimadzu SPD-M10A VP, Eagle Farm, Australia) and MAAs were identified by absorption spectra and retention time. The HPLC-based integrated absorbance was calculated based on the area under lambda maximum (λ_max_) chromatograms for isolated peaks, standardized to the dry weight of the mucus sample.

**Table 1:** Gradient protocol for eluents used in the separation of mycosporine-like amino acids on Devenosil RPAQEOUS Column.

Time (min)	Percent eluent A	Percent eluent B
0	98	2
1	98	2
5	75	25
7	60	40
12	45	55
14	40	60
16	98	2
22	98	2

Eluent A: aqueous 0.05% formic acid; eluent B: methanol with 0.05% formic acid.

## Results

### Spectral experiment

**Figure 2:**
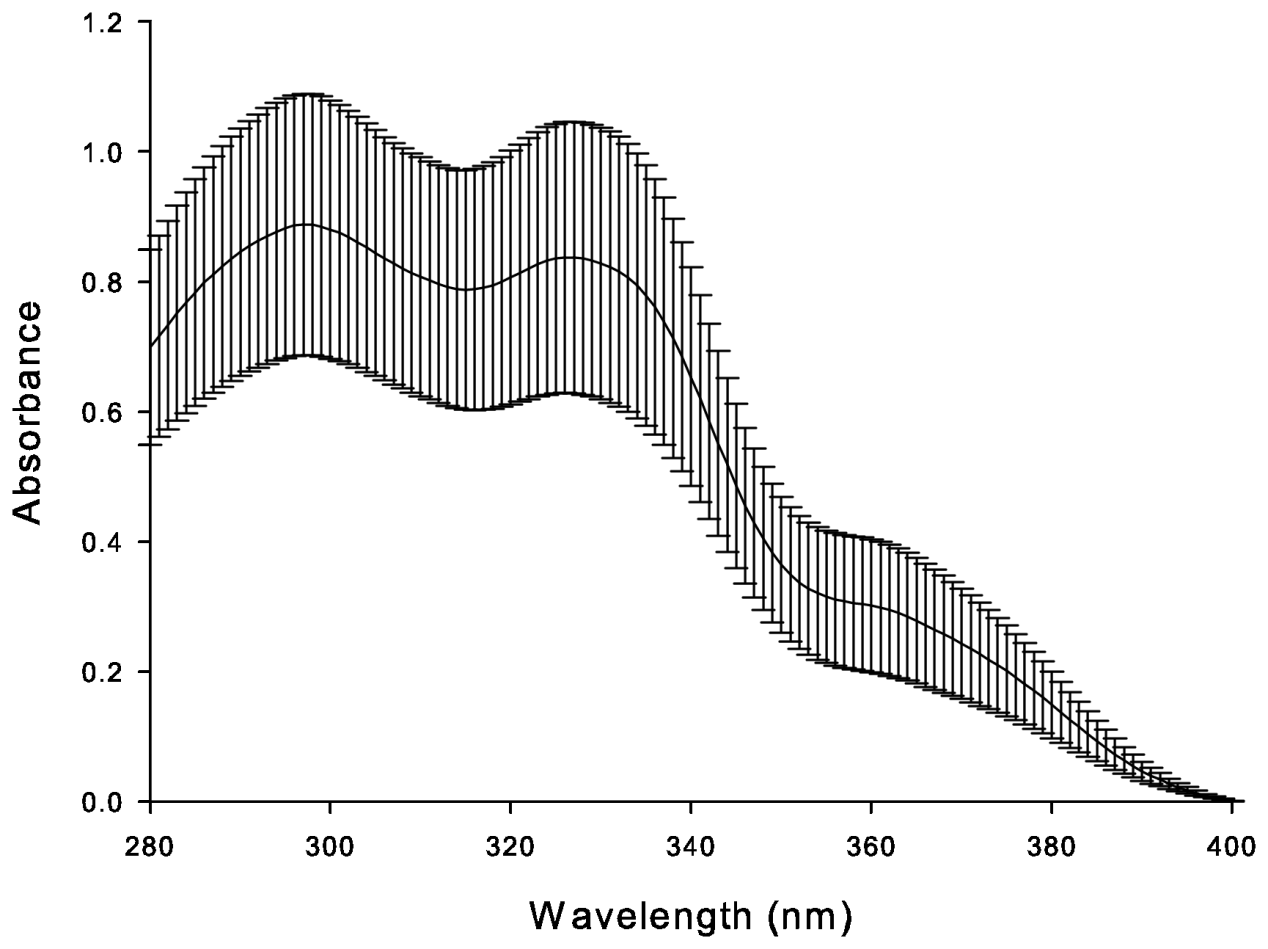
Mean (± SD) field absorbance of mucus of freshly caught cleaner fish *Labroides dimidiatus* (n = 36) from 3 m depth, according to wavelength.

The average field-measured UV absorbance of cleaner fish mucus was relatively consistent across the wavelengths sampled and showed two peaks, one at 298 nm and another at 330 nm, as well as a shoulder at 360 nm (Figure 2). 

**Figure 3:**
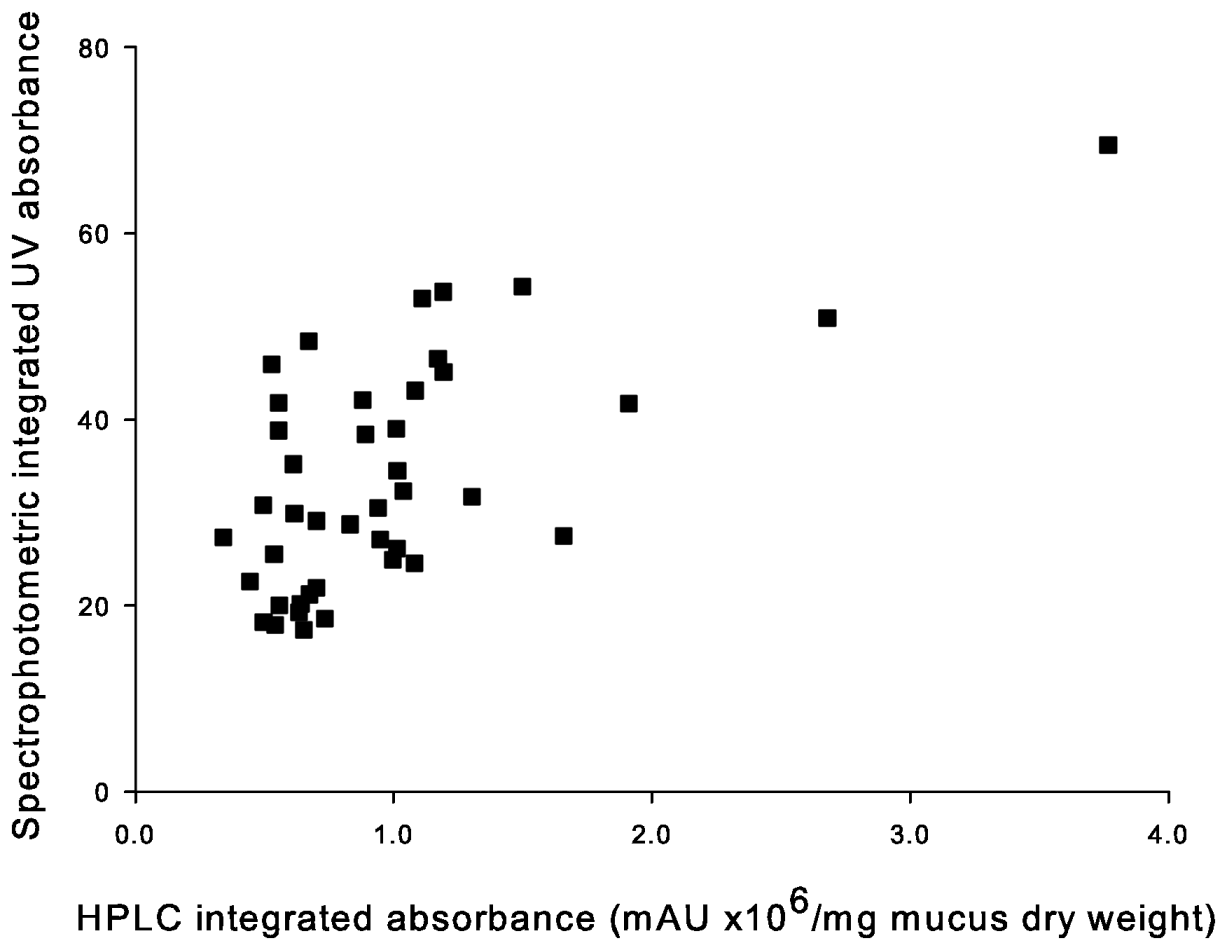
Correlation between field-measured integrated UV absorbance of cleaner fish *Labroides dimidiatus* mucus and laboratory-based mucus dry weight-standardized HPLC measurement of mycosporine-like amino acid (MAA) absorbance.

 Field-measured integrated UV absorbance of mucus measured with the USB2000 spectroradiometer correlated significantly with laboratory-measured mucus dry weight-standardized HPLC absorbance (Pearson correlation, n = 42, r = 0.64, p < 0.0001), even when the two highest “outlier” HPLC data points were omitted (n = 40, r = 0.45, p < 0.0038; Figure 3). The integrated UV absorbance of cleaner fish mucus measured immediately after capture was 68.1 ± 2.4 (mean ± SE). 

**Figure 4:**
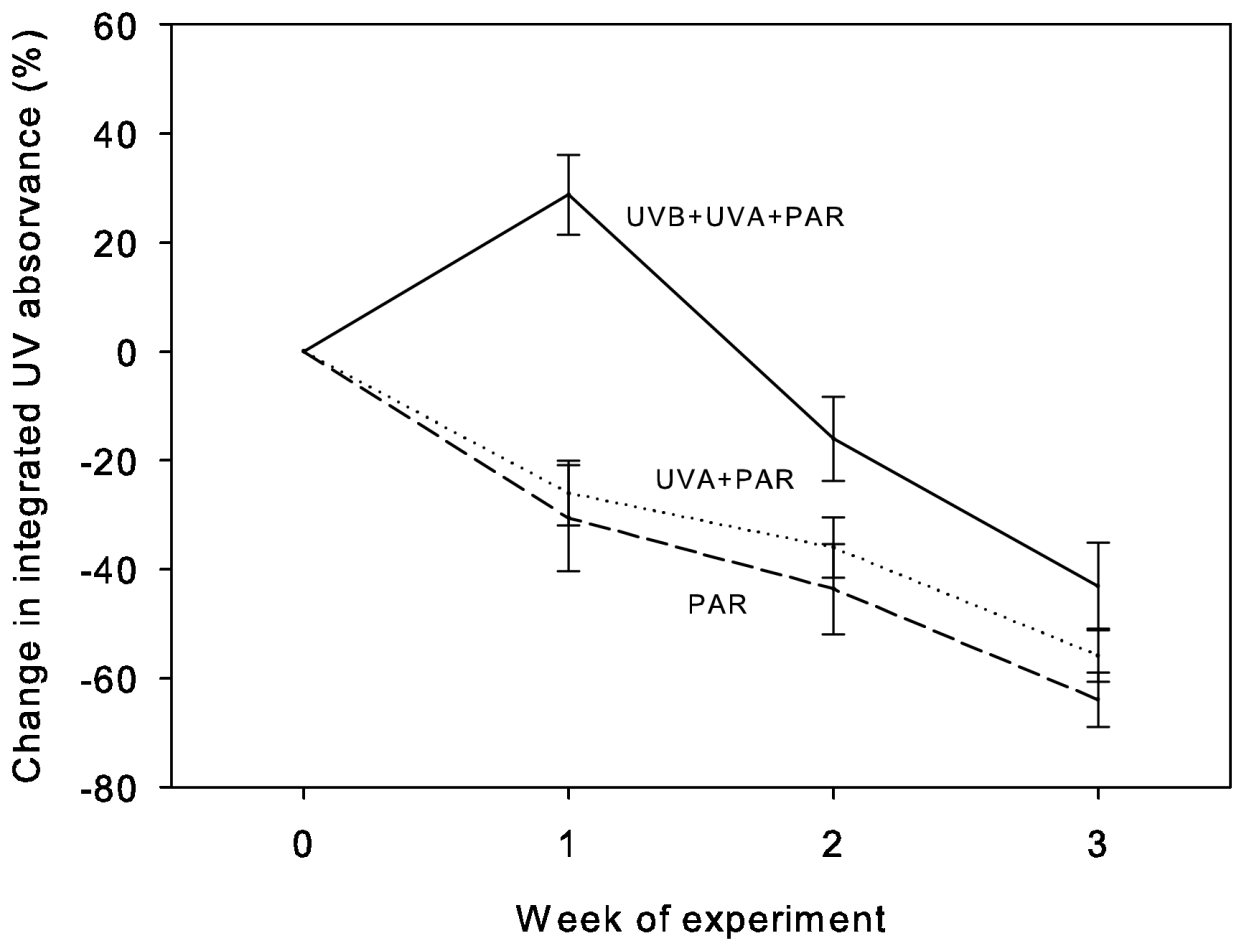
Mean (± SE) integrated UV absorbance of cleaner fish *Labroides dimidiatus* mucus in three spectral experiments over three weeks. Spectral treatments (and lines) correspond with those in Figure 1.

The analysis of the percent change in integrated UV absorbance of mucus indicated a significant interaction between the effect of UV treatment exposure and the date fish were sampled (Figure 4; Repeated Measures ANOVA: F_4,64_ = 4.76, p < 0.01). This interaction was due to the UV absorbance of mucus from the UVB+UVA+PAR treated fish being higher than the other two treatments for week one (Bonferroni-adjusted pair-wise comparisons, UVB+UVA+PAR vs. UVA+PAR: t = 5.46, p < 0.001; UVB+UVA+PAR vs. PAR: t = 5.91, p < 0.001), but not significantly different than the other treatments in weeks two and three. The remaining factors in this model were: treatment spectra exposure (F_2,34_ = 12.26, p = 0.0001), and date fish were sampled (F_2,64_ = 45.56, p < 0.0001). The diet fed to cleaners had a weight-standardized integrated HPLC absorbance of 6 AU, or 46,267 times less UV absorbance than the least absorbent mucus sample. 

**Figure 5:**
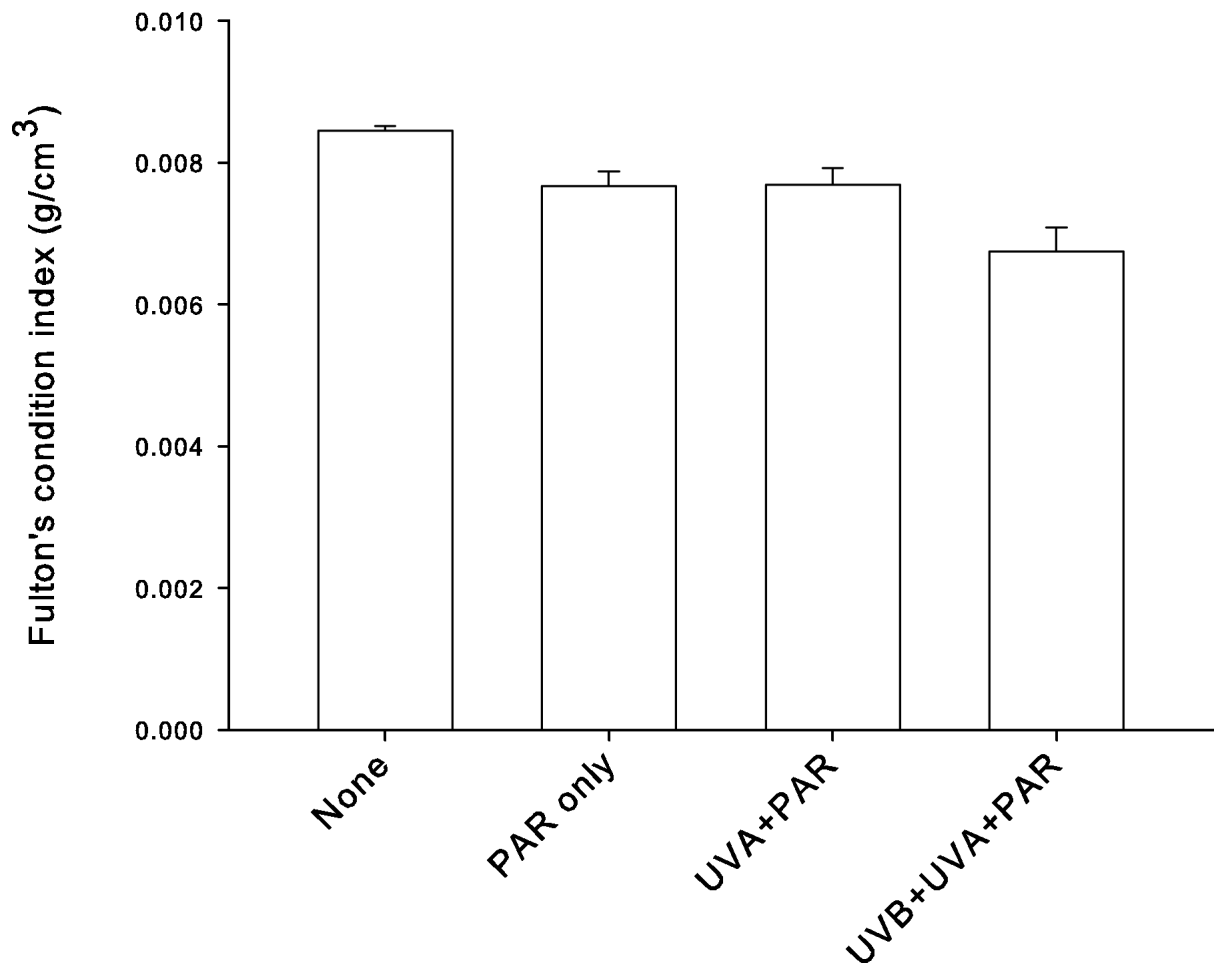
Mean (± SE) Fulton’s body condition index (K) for freshly caught cleaner fish *Labroides dimidiatus* and experimental cleaner fish after three weeks of spectral treatment. None = newly captured cleaners from 3 m depth. PAR only, UVA+PAR, and UVB+UVA+PAR are experimental treatments, which correspond with spectra in Figure 1.

 Fulton’s body condition index of fish varied significantly among treatments (Figure 5; ANOVA: F_3,85_ = 24.9, p < 0.0001). Body condition was lower for the UVB+UVA+PAR treatment group compared with the PAR and UVA+PAR treatments (Tukey post-hoc, p < 0.05), whereas the body condition of PAR treated fish was not significantly different from UVA+PAR treated fish (p > 0.05). Body condition was lower for all experimental treatment groups compared with wild-caught fish (p < 0.05).

**Figure 6:**
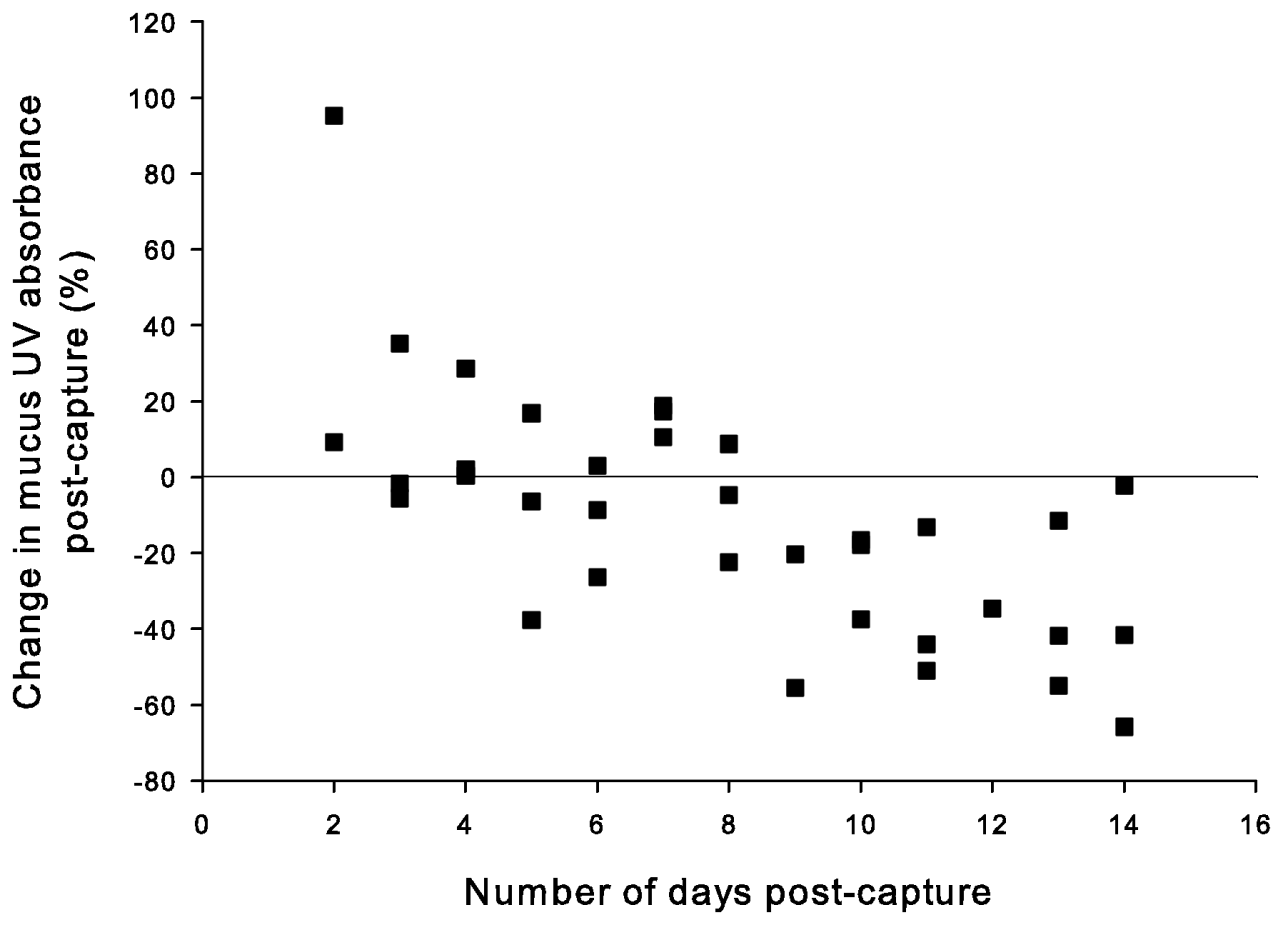
Effect of the duration of captivity on the change in UV absorbance of cleaner fish *Labroides dimidiatus* mucus. Each square represents an individual cleaner fish caught at 3 m depth.

 There was a significant effect of duration in captivity on the percent change in cleaner fish mucus absorbance (Figure 6; GLM: F_1,40_ = 5.37, p < 0.05), with all values being negative after 8 days; the effect of size of fish was not significant, nor was the interaction between duration and size significant (both p > 0.05). 

### Effect of reef depth

 The integrated UV absorbance of cleaner fish mucus increased with increasing fish length (F_1,58_ = 4.33, p = 0.04; r^2^ = 0.035), and decreased with increasing depth of capture (F_1,58_ = 53.75, p < 0.0001; r^2^ = 0.44). The average (± SE) UV absorbance decreased by more than half over the depth range studied: from 68.1 ± 2.4 at 3 m to 29.0 ± 2.7 for fish from 16 m. The magnitude of the increase in UV absorbance with length was much less, from 53.6 ± 4.4 for fish < 6.6 cm to 57.8 ± 5.5 for fish > 8.9 cm.

## Discussion

 Mucus of cleaner fish, *L. dimidiatus*, exposed to wavelengths that also included the shorter-wavelength UVB (280-700 nm) had a much higher UV absorbance after one week in captivity than did mucus of fish exposed to the spectrum that contained longer-wavelength UVA+PAR (315-700 nm), or only the spectrum visible to humans (PAR, 400-700 nm). It was known that fish could increase the level of UV absorbing compounds in their mucus when exposed to UV [20], however, this study suggests that it is the UVB portion of the spectrum that is responsible for this increase. While both UVB- and UVA-mediated induction of MAAs have been found in cyanobacteria and algae [40,41], to our knowledge this is the first time UVB-mediated MAA sequestration has been demonstrated in a higher marine organism. We know that *L. dimidiatus* are unable to visually detect differences between the treatments due to their UV-absorbing ocular media [42]. Since we found a difference between treatments, this means that the sequestering of MAAs in cleaner fish mucus is not visually mediated but must be mediated by skin or DNA damage, or some other means. 

This research also demonstrated that fish are not only able to maintain, but also to increase MAAs in the mucus for at least a week after being deprived of MAAs in the diet. As fish epithelial mucus is constantly being sloughed off and lost to the water column [17,43], MAAs must be continuously resupplied, if fish are to maintain a relatively constant level of UV absorbance of their mucus. Because their diet in the laboratory had negligible MAA content, the MAAs being supplied to the mucus likely originated from food eaten prior to capture, from some sort of “storage depot” or possibly from symbiotic bacteria. Gut evacuation in *L. dimidiatus* usually takes about 3.7 h [44]. Hence, any sequestration of MAAs into the mucus occurring beyond the first day of capture could not have directly originated from food eaten prior to capture. Instead, MAAs could have been mobilised from some sort of storage in the tissue of cleaners. Little is known about how fish process and sequester MAAs, but many invertebrates have been shown to store MAAs in various tissues. For example, sea urchins [45] and scallops [46] store MAAs in their gonads. In sea cucumbers, MAAs are present in visceral “storage depots”, but the duration of storage is unknown [47]. Similarly, sea hares store large amounts of the MAA asterina-330 in their digestive glands and these can remain for at least 40 days [48]. Fish store MAAs in their gonads [49,50], so these might serve as a potential reservoir of MAAs to be mobilised into the mucus.

Another explanation for the increase of MAAs, despite their absence in the diet, is that MAAs could have been synthesized by bacterial symbionts in the mucus (as suggested in [51]) which were subsequently affected by the lack of precursors in the experimental diet, or which subsequently compensated for the experimental UV levels in the experiment via DNA repair mechanisms. There is, however, evidence that fish mucus possesses strong antibacterial properties, at least against some bacterial species (e.g., [52,53]). Both preceding explanations must be considered highly speculative at this point, and future studies are required to investigate the mechanism underlying the temporarily increased MAA presence in the mucus of cleaner fish exposed to UVB radiation, in the absence of MAA-rich food. 

One clue to the length of time that dietary MAAs may be stored by cleaner fish was the significant interaction between UV treatments and week sampled in the spectral experiment. This was due to the difference between the UVB+UVA+PAR treatment and the other treatments after the first week of exposure that was no longer detectable after two and three weeks, suggesting that within 14 days fish exhausted any stored MAA supply. Our duration in captivity experiment suggests that, if MAA storage occurs, it is over a shorter time period; specifically, field-captured cleaner fish were able to increase the UV absorbance of their mucus for a maximum of eight days before depleting the available supply. 

 Cleaner fish exposed to the UVB+UVA+PAR treatment had a lower Fulton’s body condition index compared with fish from the other treatments. Given that experimental fish did not change length appreciably during the 3 weeks of the experiment, the decrease in this condition index (fish weight/fish standard length^3^) is due to a decrease in fish weight, and thus indicates an energetic cost. The slight, but significant, difference in body condition between wild cleaner fish and fish from all other treatment (captive) groups that we found suggests that there was such a cost to living in captivity. Even so, UVB+UVA+PAR treated cleaner fish had a significantly lower body condition than UVA+PAR or PAR treated fish. This may be due to an energetic cost of MAA sequestration, as the UVB+UVA+PAR treatment group was the only one to increase MAA sequestration while in captivity. Support for this idea is that, in Hawaii, experimental fish provided with dietary MAAs, but not exposed to UV, did not sequester MAAs in their mucus, presumably due to some energetic cost of sequestration [20]. This could also be due to a costly damage to the immune system, or the cost of photorepair of UVB-induced cellular damage. Indeed, exposure to UVB affects fish immune systems [4], reducing hematocrit, plasma protein, and plasma immunoglobulin levels while altering the function of head, kidney and blood phagocytes [54]. While Zamzow [20] found skin lesions and other signs of UV damage in fish exposed to high levels of solar UVA+PAR and UVB+UVA+PAR in Hawaii, and, despite the fact that Lizard Island is closer to the equator and thus experiences higher UV levels than Hawaii, we saw no gross evidence of skin damage. This suggests that cleaners may have pathways to repair UVB-induced damage, for example by UVA/blue light mediated photo-repair, such as is found in other fishes (e.g. zebrafish [2] and Antarctic fish larvae and eggs [5]). Repairing UVB-induced damage would likely come at an energetic cost and thus could also have contributed to the loss in weight that fish experienced. 

 UV absorbance measured via spectrophotometry in the field correlated significantly with laboratory-based HPLC results, demonstrating that both techniques reflect the actual concentrations of MAAs in the mucus. HPLC analyses are orders of magnitude more time-consuming, expensive, and equipment-intensive than spectrophotometry. While HPLC is a superior technique for investigations of the precise composition and quantity of MAAs in the mucus, we suggest that field absorbance measures may be sufficient if one desires simply to measure overall UV absorbance without consideration of the specific compounds causing the absorbance. We found three peaks (298, 330 and 360 nm) in our field measurements of the absorbance of mucus of the freshly caught *L. dimidiatus*. Two peaks correlate with the lambda maxima of the MAAs asterina-330 (330 nm peak) and palythene (360 nm peak) which have been identified in cleaner fish mucus from Heron Island, GBR [18]. Gadusol and deoxygadusol, two compounds found in fish and invertebrate eggs [49,50,55] are known to have lambda maxima of 296 nm and 294 nm [55], respectively, but these compounds have not been detected in *L. dimidiatus* mucus by HPLC [18], so the source of the 298 nm peak is unclear. In addition to MAAs, fish mucus also contains various proteins, amino acids and other compounds, which contributed to the field measured integrated absorbance values found in this study [56,57].

 The average integrated UV absorbance of wild-caught cleaner fish was similar to that found in *L. dimidiatus* by Eckes et al. [18] and it was 1.6 and 2.4 times higher than values previously reported for another labrid, *Thalassoma duperrey*, and a pomacentrid, *Pomacentrus amboinensis*, respectively [20,58]. Zamzow (2007) used methods identical to those in this study to measure the mucus absorbance of the labrid *Halichoeres bivittatus* from clear Panamanian waters at shallow depths, and found peak absorbance values of less than half those of *L. dimidiatus* [22].This is consistent with the finding that *L. dimidiatus* possess large amounts of the MAAs asterina-330 and palythene in their mucus [18]. As fishes are unable to synthesize MAAs due to the absence of the shikimate pathway in higher metazoans ([59], but see 60), cleaner fish must acquire these compounds from their diet (i.e. parasites or mucus of client fish [29]). When given a choice between parasitic gnathiid isopods, the main component of their diet in the wild [29], and parrotfish *Chlorurus sordidus* mucus, *L. dimidiatus* preferred the mucus [31]. Furthermore, when offered two types of fish mucus, cleaner fish preferred the mucus of *C. sordidus* to that of the snapper *Lutjanus fulviflamma* [61]. While such variation in dietary preferences in cleaner fish will affect the mutualistic outcome of the cleaning interactions between them and their clients, it also raises the question of whether their preferences are influenced by the MAA levels of these foods or some other nutritional or energetic aspect. *C. sordidus* has very high levels of MAAs in the mucus, the highest of five parrotfish species sampled by Eckes et al. [18], and *L. fulviflamma* has orders of magnitude less UV absorbent mucus [62], yet both species appear to occur at similar depths (A.S.G. pers. obs.). Nutritionally, *C. sordidus* mucus is also superior to that of *L. fulviflamma* [62].

 Even though the size range of fish in this study was relatively small (3.9 cm), and the relationship of UV absorbance with size fairly weak in comparison to the relationship with depth, the mucus of large cleaner fish had a greater UV absorbance than that of smaller fish. *L. dimidiatus* have a dominance hierarchy based on size [63]. Thus larger individuals are likely better competitors for clients and so might maximize MAAs through competition with smaller fish for client fish mucus with better sources of MAAs. A much stronger positive correlation between size and mucus absorbance (r^2^ = 0.66) was found for a pomacentrid, *P. amboinensis*, over a size range of 5.8 cm [58]. However, in a similar study with the labrid *T. duperrey*, Zamzow [20] found an effect of sex, but not size, on the UV absorbance of mucus. *L. dimidiatus*, also a labrid, is a protogynous sex changer and normally changes from female to male, but can, on rare occasions, also change from male to female, depending on the size of conspecifics present [64]. The largest fish in a social group, however, is always a male [64]. Thus, the observed relationship with size may be confounded with sex. As we did not sacrifice the fish, we were unable to determine the sex of our study animals, and cannot rule out a possible effect of sex on mucus MAA concentration for this species. 

 Decreases in the amount of UV absorbed by fish mucus with increasing depth have previously been demonstrated for *T. duperrey* in relatively turbid Hawaiian waters [17]. Here, we found a similar correlation for *L. dimidiatus* in the clear waters surrounding Lizard Island. Such a decrease in UV absorbance with depth might be due to control of the MAA concentration of the mucus by the cleaners, or due to a lack of availability of UV absorbing compounds in their food supply. Our spectral experiments suggest that the observed changes in UV absorbance may be controlled by UVB exposure. Cleaners in the UVB+UVA+PAR treatment received the same amount of MAAs in their diet as the fish in the other two treatments but they had a markedly higher level of epithelial UV absorbance, at least initially. However, even under UVB+UVA+PAR exposure, the cleaners could not maintain elevated concentrations of MAAs in the mucus indefinitely due to a dietary insufficiency of the requisite MAAs. Thus, we cannot rule out dietary insufficiency, due to decreasing amounts of MAAs in the food with depth, as a reason for decreased mucus absorbance with depth. Whatever is driving the relationship, the correlation of UV absorbance of the mucus with capture depth is clear. While we did not simultaneously catch fish and measure UV radiation during this experiment, the fact that UV radiation attenuates with depth is well known [24]. Likewise, while we do not know the effective dose of UV radiation received by cleaner fish individuals while at various depths, our interest was in the potential relative effects of capture depth on mucus absorbance, and our correlation suggests a strong relationship.

UVB exposure levels in the tropics are naturally amongst the highest on Earth, and have been gradually increasing at our study site [11]. In the face of such pressures, the ability of cleaner fish and other fishes to adjust their mucus UV protection in response to environmental UVB exposure, whether by selecting food higher in MAAs or by physiological adaptations to maximize MAA secretion, may prove valuable. 

## References

[1] Kligman LH, Akin FJ, Kligman AM (1985) The contributions of UVA and UVB to connective-tissue damage in hairless mice. J Invest Dermatol 84: 272-276. doi:10.1111/1523-1747.ep12265353. PubMed: 3981040. 3981040

[2] Dong Q, Svoboda K, Tiersch TR, Monroe WT (2007) Photobiological effects of UVA and UVB light in zebrafish embryos: Evidence for a competent photorepair system. J Photochem Photobiol B Biol 88: 137-146. doi:10.1016/j.jphotobiol.2007.07.002. PubMed: 17716904. PMC5600543 17716904

[3] Lesser MP, Farrell JH, Walker CW (2001) Oxidative stress, DNA damage and p53 expression in the larvae of Atlantic cod (*Gadus* *morhua*) exposed to ultraviolet (290-400 nm) radiation. J Exp Biol 204: 157-164. PubMed: 11104719. 11104719 10.1242/jeb.204.1.157

[4] Markkula SE, Salo HM, Rikalainen AK, Jokinen EI (2006) Different sensitivity of carp (*Cyprinus* *carpio*) and rainbow trout (*Oncorhynchus* *mykiss*) to the immunomodulatory effects of UVB irradiation. Fish Shellfish Immunol 21: 70-79. doi:10.1016/j.fsi.2005.10.007. PubMed: 16376572. 16376572

[5] Malloy KD, Holman MA, Mitchell D, Detrich HW 3rd (1997) Solar UVB-induced DNA damage and photoenzymatic DNA repair in antarctic zooplankton. Proc Natl Acad Sci USA 94: 1258-1263. doi:10.1073/pnas.94.4.1258. PubMed: 9037040. 9037040 PMC19778

[6] Ahmed FE, Setlow RB (1993) Ultraviolet radiation-induced DNA damage and its photorepair in the skin of the platyfish *Xiphophorus* . Cancer Res 53: 2249-2255. PubMed: 8485710. 8485710

[7] Banaszak AT, Lesser MP (2009) Effects of solar ultraviolet radiation on coral reef organisms. Photochem Photobiol Sci 8: 1276-1294. doi:10.1039/b902763g. PubMed: 19707616. 19707616

[8] Frederick JE, Snell HE, Haywood EK (1989) Solar ultraviolet-radiation at the Earth's surface. Photochem Photobiol 50: 443-450. doi:10.1111/j.1751-1097.1989.tb05548.x.

[9] McKenzie RL, Aucamp PJ, Bais AF, Björn LO, Ilyas M (2007) Changes in biologically-active ultraviolet radiation reaching the Earth's surface. Photochem Photobiol Sci 6: 218-231. doi:10.1039/b700017k. PubMed: 17344959. 17344959

[10] Wild M, Gilgen H, Roesch A, Ohmura A, Long CN et al. (2005) From dimming to brightening: decadal changes in solar radiation at Earth's surface. Science 308: 847-850. doi:10.1126/science.1103215. PubMed: 15879214. 15879214

[11] Masiri I, Nunez M, Weller E (2008) A 10-year climatology of solar radiation for the Great Barrier Reef: implications for recent mass coral bleaching events. Int J Remote Sens 29: 4443-4462. doi:10.1080/01431160801930255.

[12] Baker KS, Smith RC (1982) Bio-optical classification and model of natural waters. Limnol Oceanogr 27: 500-509. doi:10.4319/lo.1982.27.3.0500.

[13] Dunlap WC, Shick JM, Yamamoto Y (2000) UV protection in marine organisms. I. Sunscreens, oxidative stress and antioxidants. In: Yoshikawa T Toyokuni S Yamamoto Y Naito Y Free radicals in chemistry, biology and medicine: OICA. International: 200-214.

[14] Frank TM, Widder EA (1996) UV light in the deep-sea: *in* *situ* measurements of downwelling irradiance in relation to the visual threshold sensitivities of UV-sensitive crustaceans. Mar Freshwat Behav Physiol 27: 189-197. doi:10.1080/10236249609378964.

[15] Sweet M, Kirkham N, Bendall M, Currey L, Bythell J et al. (2012) Evidence of melanoma in wild marine fish populations. PLOS ONE 7: e41989. doi:10.1371/journal.pone.0041989. PubMed: 22870273. 22870273 PMC3411568

[16] Dunlap WC, Williams DM, Chalker BE, Banaszak AT (1989) Biochemical photoadaptation in vision: U.V.-absorbing pigments in fish eye tissues. Comp Biochem Physiol B Biochem Mol Biol 93: 601-607.

[17] Zamzow JP, Losey GS (2002) Ultraviolet radiation absorbance by coral reef fish mucus: photo-protection and visual communication. Environ Biol Fishes 63: 41-47. doi:10.1023/A:1013846816869.

[18] Eckes MJ, Siebeck UE, Dove S, Grutter AS (2008) Ultraviolet sunscreens in reef fish mucus. Mar Ecol Prog Ser 353: 203-211. doi:10.3354/meps07210.

[19] Mason DS, Schafer F, Shick JM, Dunlap WC (1998) Ultraviolet radiation-absorbing mycosporine-like amino acids (MAAs) are acquired from their diet by medaka fish (*Oryzias* *latipes*) but not by SKH-1 hairless mice. Comp Biochem Physiol A 120: 587-598. doi:10.1016/S0305-0491(98)10051-2. 9828392

[20] Zamzow JP (2004) Effects of diet, ultraviolet exposure, and gender on the ultraviolet absorbance of fish mucus and ocular structures. Mar Biol 144: 1057-1064. doi:10.1007/s00227-003-1286-2.

[21] Zamzow JP (2003) Ultraviolet-absorbing compounds in the mucus of temperate Pacific tidepool sculpins: variation over local and geographic scales. Mar Ecol Prog Ser 263: 169-175. doi:10.3354/meps263169.

[22] Zamzow JP (2007) Ultraviolet-absorbing compounds in the mucus of shallow-dwelling tropical reef fishes correlate with environmental water clarity. Mar Ecol Prog Ser 343: 263-271. doi:10.3354/meps06890.

[23] Zamzow JP, Nelson PA, Losey GS (2008) UV lights up marine fish. Am Sci 96: 482-489. doi:10.1511/2008.75.482.

[24] Jerlov NG (1976) Marine optics. Amsterdam, NY: Elsevier Scientific. 231 pp.

[25] Dunlap WC, Chalker BE, Oliver JK (1986) Bathymetric adaptations of reef-building corals at Davies Reef, Great-Barrier-Reef, Australia. 3. UV-B absorbing compounds. J Exp Mar Biol Ecol 104: 239-248. doi:10.1016/0022-0981(86)90108-5.

[26] Karentz D, Dunlap WC, Bosch I (1997) Temporal and spatial occurrence of UV-absorbing mycosporine-like amino acids in tissues of the Antarctic sea urchin *Sterechinus* *neumayeri* during springtime ozone-depletion. Mar Biol 129: 343-353. doi:10.1007/s002270050174.

[27] Karsten U, Sawall T, Hanelt D, Bischof K, Figueroa FL et al. (1998) An inventory of UV-absorbing mycosporine-like amino acids in macroalgae from polar to warm-temperate regions. Bot 3 41: 443-453.

[28] Shick JM, Dunlap WC, Chalker BE, Banaszak AT, Rosenzweig TK (1992) Survey of ultraviolet radiation-absorbing mycosporine-like amino-acids in organs of coral-reef holothuroids. Mar Ecol Prog Ser 90: 139-148. doi:10.3354/meps090139.

[29] Grutter AS (1997) Spatiotemporal variation and feeding selectivity in the diet of the cleaner fish *Labroides* *dimidiatus* . Copeia: 346-355.

[30] Bshary R (2003) The cleaner wrasse, *Labroides* *dimidiatus*, is a key organism for reef fish diversity at Ras Mohammed National Park, Egypt. J Anim Ecol 72: 169-176. doi:10.1046/j.1365-2656.2003.00683.x.

[31] Grutter AS, Bshary R (2003) Cleaner wrasse prefer client mucus: support for partner control mechanisms in cleaning interactions. Proc R Soc Lond B Biol Sci 270: S242-S244. doi:10.1098/rsbl.2003.0077. PubMed: 14667394. PMC1809949 14667394

[32] Fulton CJ, Bellwood DR, Wainwright PC (2001) The relationship between swimming ability and habitat use in wrasses (Labridae). Mar Biol 139: 25-33. doi:10.1007/s002270100565.

[33] Grutter AS (1995) Relationship between cleaning rates and ectoparasite loads in coral-reef fishes. Mar Ecol Prog Ser 118: 51-58. doi:10.3354/meps118051.

[34] Ricker WE (1975) Computation and interpretation of biological statistics of fish populations 191 Bull Fish Res Board Canada pp. 1-382. PubMed: 1110344.

[35] Banaszak AT, Lesser MP, Kuffner IB, Ondrusek M (1998) Relationship between ultraviolet (UV) radiation and mycosporine-like amino acids (MAAS) in marine organisms. Bull Mar Sci 63: 617-628.

[36] Carroll AK, Shick JM (1996) Dietary accumulation of UV-absorbing mycosporine-like amino acids (MAAs) by the green sea urchin (*Strongylocentrotus* *droebachiensis*). Mar Biol 124: 561-569. doi:10.1007/BF00351037.

[37] Dunlap WC, Shick JM (1998) Ultraviolet radiation-absorbing mycosporine-like amino acids in coral reef organisms: A biochemical and environmental perspective. J Phycol 34: 418-430. doi:10.1046/j.1529-8817.1998.340418.x.

[38] Jeffrey SW, MacTavish HS, Dunlap WC, Vesk M, Groenewoud K (1999) Occurrence of UVA- and UVB-absorbing compounds in 152 species (206 strains) of marine microalgae. Mar Ecol Prog Ser 189: 35-51. doi:10.3354/meps189035.

[39] Karentz D, Mceuen FS, Land MC, Dunlap WC (1991) Survey of mycosporine-like amino-acid compounds in Antarctic marine organisms - potential protection from ultraviolet exposure. Mar Biol 108: 157-166. doi:10.1007/BF01313484.

[40] Kräbs G, Watanabe M, Wiencke C (2004) A monochromatic action spectrum for the photoinduction of the UV-absorbing mycosporine-like amino acid shinorine in the red alga *Chondrus* *crispus* . Photochem Photobiol 79: 515-519. doi:10.1562/2003-12-14-RA.1. PubMed: 15291302. 15291302

[41] Portwich A, Garcia-Pichel F (2000) A novel prokaryotic UVB photoreceptor in the cyanobacterium *Chlorogloeopsis* PCC 6912. Photochem Photobiol 71: 493-498. doi:10.1562/0031-8655(2000)071. PubMed: 10824604. 10824604

[42] Siebeck UE, Marshall NJ (2001) Ocular media transmission of coral reef fish - can coral reef fish see ultraviolet light? Vision Res 41: 133-149. doi:10.1016/S0042-6989(00)00240-6. PubMed: 11163849. 11163849

[43] Shephard KL (1994) Functions for fish mucus. Rev Fish Biol Fish 4: 401-429. doi:10.1007/BF00042888.

[44] Grutter AS (1996) Parasite removal rates by the cleaner wrasse *Labroides* *dimidiatus* . Mar Ecol Prog Ser 130: 61-70. doi:10.3354/meps130061.

[45] Gravem SA, Adams NL (2012) Sex and microhabitat influence the uptake and allocation of mycosporine-like amino acids to tissues in the purple sea urchin, *Strongylocentrotus* *purpuratus* . Mar Biol 159: 2839-2852. doi:10.1007/s00227-012-2045-z.

[46] Oyamada C, Kaneniwa M, Ebitani K, Murata M, Ishihara K (2008) Mycosporine-like amino acids extracted from scallop (*Patinopecten* *yessoensis*) ovaries: UV protection and growth stimulation activities on human cells. Mar Biotechnol ( NY) 10: 141-150 doi:10.1007/s10126-007-9043-z. PubMed: 18157682. 18157682

[47] Bandaranayake WM, Des Rocher A (1999) Role of secondary metabolites and pigments in the epidermal tissues, ripe ovaries, viscera, gut contents and diet of the sea cucumber *Holothuria* *atra* . Mar Biol 133: 163-169. doi:10.1007/s002270050455.

[48] Carefoot TH, Harris M, Taylor BE, Donovan D, Karentz D (1998) Mycosporine-like amino acids: possible UV protection in eggs of the sea hare *Aplysia* *dactylomela* . Mar Biol 130: 389-396. doi:10.1007/s002270050259.

[49] Chioccara F, Dellagala A, Derosa M, Novellino E, Prota G (1980) Mycosporine amino-acids and related-compounds from the eggs of fishes. Bull Soc Chim Bel 89: 1101-1106.

[50] Arbeloa EM, Uez MJ, Bertolotti SG, Churio MS (2010) Antioxidant activity of gadusol and occurrence in fish roes from Argentine Sea. Food Chem 119: 586-591. doi:10.1016/j.foodchem.2009.06.061.

[51] Carreto JI, Carignan MO (2011) Mycosporine-like amino acids: Relevant secondary metabolites. Chemical and ecological aspects. Mar Drugs 9: 387-446. doi:10.3390/md9030387. PubMed: 21556168. 21556168 PMC3083659

[52] Ebran N, Julien S, Orange N, Saglio P, Lemaître C et al. (1999) Pore-forming properties and antibacterial activity of proteins extracted from epidermal mucus of fish. Comp Biochem Physiol A 122: 181-189. doi:10.1016/S0305-0491(98)10157-8. PubMed: 10327617. 10327617

[53] Subramanian S, Ross NW, MacKinnon SL (2008) Comparison of antimicrobial activity in the epidermal mucus extracts of fish. Comp Biochem Physiol B_Biochem Mol Biol 150: 85-92. doi:10.1016/j.cbpa.2008.04.151. PubMed: 18342561. 18342561

[54] Salo HM, Jokinen EI, Markkula SE, Aaltonen TM, Penttilä HT (2000) Comparative effects of UVA and UVB irradiation on the immune system of fish. J Photochem Photobiol B Biol 56: 154-162. doi:10.1016/S1011-1344(00)00072-5. PubMed: 11079476. 11079476

[55] Plack PA, Fraser NW, Grant PT, Middleton C, Mitchell AI et al. (1981) Gadusol, an enolic derivative of cyclohexane-1,3-dione present in the roes of cod and other marine fish - isolation, properties and occurrence compared with ascorbic-acid. Biochem J 199: 741-747. PubMed: 7200360. 7200360 10.1042/bj1990741 PMC1163432

[56] Chong K, Ying TS, Foo J, Jin LT, Chong A (2005) Characterisation of proteins in epidermal mucus of discus fish (*Symphysodon* spp.) during parental phase. Aquaculture 249: 469-476. doi:10.1016/j.aquaculture.2005.02.045.

[57] Wessler E, Werner I (1957) On the chemical composition of some mucous substances of fish. Acta Chem Scand 11: 1240-1247. doi:10.3891/acta.chem.scand.11-1240.

[58] Zamzow JP, Siebeck UE (2006) Ultraviolet absorbance of the mucus of a tropical damselfish: effects of ontogeny, captivity and disease. J Fish Biol 69: 1583-1594. doi:10.1111/j.1095-8649.2006.01219.x.

[59] Bentley R (1990) The Shikimate pathway - a metabolic tree with many branches. Crit Rev Biochem Mol Biol 25: 307-384. doi:10.3109/10409239009090615. PubMed: 2279393. 2279393

[60] Shinzato C, Shoguchi E, Kawashima T, Hamada M, Hisata K et al. (2011) Using the *Acropora* *digitifera* genome to understand coral responses to environmental change. Nature 476: 320-323. doi:10.1038/nature10249. PubMed: 21785439. 21785439

[61] Grutter AS, Bshary R (2004) Cleaner fish, *Labroides* *dimidiatus*, diet preferences for different types of mucus and parasitic gnathiid isopods. Anim Behav 68: 583-588. doi:10.1016/j.anbehav.2003.11.014.

[62] Eckes MJ (2009) The ecological function of fish mucus [PhD]. The University of Queensland. 192 pp.

[63] Robertson DR (1972) Social control of sex reversal in a coral-reef fish. Science 177: 1007–1009. doi:10.1126/science.177.4053.1007. PubMed: 17788814. 17788814

[64] Kuwamura T, Tanaka N, Nakashima Y, Karino K, Sakai Y (2002) Reversed sex-change in the protogynous reef fish *Labroides* *dimidiatus* . Ethology 108: 443-450. doi:10.1046/j.1439-0310.2002.00791.x.

